# Molecular analysis of genetic mutations in non-small cell lung cancer in Morocco

**DOI:** 10.11604/pamj.2024.47.116.42973

**Published:** 2024-03-11

**Authors:** Ouafaa Morjani, Nouhad Benkirane, Hassan Errihani, El Mostafa Elfahime, Hamid Lakhiari

**Affiliations:** 1 Laboratory of Virology, Oncology, Biosciences, Environment, and New Energies, Faculty of Sciences and Technics Mohammedia, Hassan II University Casablanca Morocco, Mohammedia, Morocco,; 2 Pathology Laboratory of the Center, Mohamed Zerktouni, Casablanca, Morocco,; 3 National Institute of Oncology, Av. Allal Al Fassi, Rabat, Morocco,; 4 Functional Genomic Platform, National Center of Scientific and Technical Research, Allal El Fassi, Hay Ryad, Rabat, Morocco

**Keywords:** Non-small cell lung cancer, adenocarcinoma, genetic mutations, Epidermal Growth Factor Receptor, Moroccan population

## Abstract

Non-small cell lung cancer (NSCLC) is a significant global health issue with diverse molecular profiles affecting treatment responses. Yet, NSCLC's molecular epidemiology in Morocco is largely unexplored. This study focuses on NSCLC genetic mutations, specifically in adenocarcinoma, among Moroccan patients to contribute to understanding NSCLC in this population. Ninety-four patients diagnosed with lung adenocarcinoma were analyzed. Formalin-fixed paraffin-embedded tissue samples were processed, and deoxyribonucleic acid (DNA)/ribonucleic acid (RNA) was extracted using standardized protocols. Mutations were detected using the AmoyDx Pan Lung Cancer Polymerase Chain Reaction (PCR) Panel kit, and their frequencies were assessed through statistical analysis. Epidermal Growth Factor Receptor (EGFR) mutations were detected in 22.34% of patients, predominantly exon 19 deletions (66.66%) and exon 21 L858R mutations (23.80%). Anaplastic lymphoma kinase (ALK) gene fusion was observed in 3.19% of patients, and KRAS mutations in 1.06%. No mutations were found in other tested genes. A slightly higher mutation rate was noted in females (54.16%) compared to males (45.84%). The study reveals a distinct mutation profile in Moroccan NSCLC patients, with a notable prevalence of EGFR mutations, albeit lower than in some Asian populations. The significance of EGFR mutations in treatment response aligns with global findings, highlighting the importance of understanding regional molecular variations for personalized therapy. Despite limitations in sample size and clinical data, this study sheds light on the genetic landscape of NSCLC in Morocco. The observed mutation rates, particularly in EGFR, underscore the potential for targeted therapies in Moroccan NSCLC patients, emphasizing the need for further research to refine treatment strategies tailored to this population.

## Introduction

Lung cancer poses a significant public health challenge and remains one of the foremost global causes of cancer-related mortality, with over 2.2 million new cases diagnosed annually and approximately 1.8 million deaths [1]. Non-small cell lung cancer (NSCLC) comprises the predominant subtype, accounting for approximately 85% of lung cancer cases [2]. This category encompasses adenocarcinoma, the most prevalent histological type, representing over 50% of cases [3], as well as squamous cell carcinoma, large cell carcinoma, adenosquamous carcinoma, mucoepidermoid carcinoma, and sarcomatoid carcinoma. Presently, the standard approach for managing lung adenocarcinoma involves early surgical intervention and chemotherapy for advanced stages. However, due to insufficient screening awareness and outdated diagnostic techniques, the majority of patients receive their diagnosis at an advanced stage, resulting in diminished overall survival rates. Consequently, recent advances in genomics have wrought a revolution in our understanding of gene functions and signaling mechanisms associated with lung adenocarcinoma, thereby opening up new vistas for molecular diagnosis and targeted therapeutic strategies, facilitating early disease detection [4]. The National Comprehensive Cancer Network (NCCN) has also underscored the importance of ascertaining the genetic mutation status in NSCLC patients to enhance the efficacy of targeted therapies [5]. Nonetheless, our comprehension of the molecular epidemiology of lung cancer within the Moroccan population remains circumscribed.

The objective of this study is to scrutinize the mutational status of driver genes implicated in NSCLC within a cohort of Moroccan patients afflicted with lung adenocarcinoma. On the foundation of our detection efforts, we have sought to identify the most prevalent mutations and assess the various permutations thereof. These findings can serve to augment the body of knowledge pertaining to the mutational status of NSCLC in Morocco. Furthermore, they establish a reference framework for prospective investigations into the associations between genetic mutations and the clinical characteristics of Moroccan NSCLC patients. This study contributes significantly to the burgeoning field of molecular oncology and has the potential to drive improvements in the diagnosis and treatment of NSCLC in the Moroccan context.

## Methods

Ninety-four cases of patients diagnosed with lung adenocarcinoma by skilled cytologists, with subsequent molecular analysis, were collected between January 2023 and June 2023 at the Center Pathology Laboratory.

**Sample processing:** all samples analyzed in this study were formalin-fixed and paraffin-embedded tissues. Adequate tissue sections were taken and placed in 1.5 mL tubes. These tubes were incubated at 56°C for 3 minutes after adding 1 mL of xylene and thorough vortexing. Subsequently, the tubes were centrifuged at 13,000 xg for 2 minutes at room temperature to separate the supernatant from the precipitate, which was removed by pipetting while avoiding contact with the precipitate. To eliminate any remaining paraffin residue, 1 mL of 96% ethanol was added, and the tubes were vortexed and centrifuged at 13,000 xg for 2 minutes at room temperature. The supernatant was again removed by pipetting without touching the precipitate. Finally, the tube was left open to allow the precipitate to air-dry at 56°C for a duration ranging from 1 to 10 minutes until the tissue had a matte surface.

**DNA and RNA extraction:** DNA and RNA were extracted from the paraffin-embedded samples following the instructions provided with the AmoyDx FFPE DNA/RNA Kit (AmoyDx Inc, Xiamen, China). The extracted DNA was stored at -20°C in a refrigerator, while the RNA was stored at -70°C for subsequent use.

**Detection of genetic mutations:** for mutation detection, the AmoyDx Pan Lung Cancer PCR Panel kit was utilized. This is a real-time PCR test designed for the qualitative detection of 167 common alterations in the EGFR, ALK, ROS1, KRAS, BRAF, HER2, RET, MET, NTRK1, NTRK2, and NTRK3 genes in NSCLC patients. The kit includes an RNA gene fusion detection system in reaction mix A (ALK, ROS1, RET, MET, NTRK1, NTRK2, and NTRK3), a DNA gene mutation detection system in reaction mix B (EGFR, KRAS, BRAF, and HER2), a positive control, and a negative control. This genetic mutation detection system utilizes AmoyDx Amplification Refractory Mutation System (ADx-ARMS) technology, comprising specific primers and fluorescent probes to detect genetic mutations.

**Statistical analysis:** data entry was performed using Microsoft Office Excel 2016. Statistical analysis was carried out using Microsoft Excel 16.16.27 and the Jamovi software. Quantitative variables were represented using counts (N) and percentages (%).

## Results

**Figure 1:**
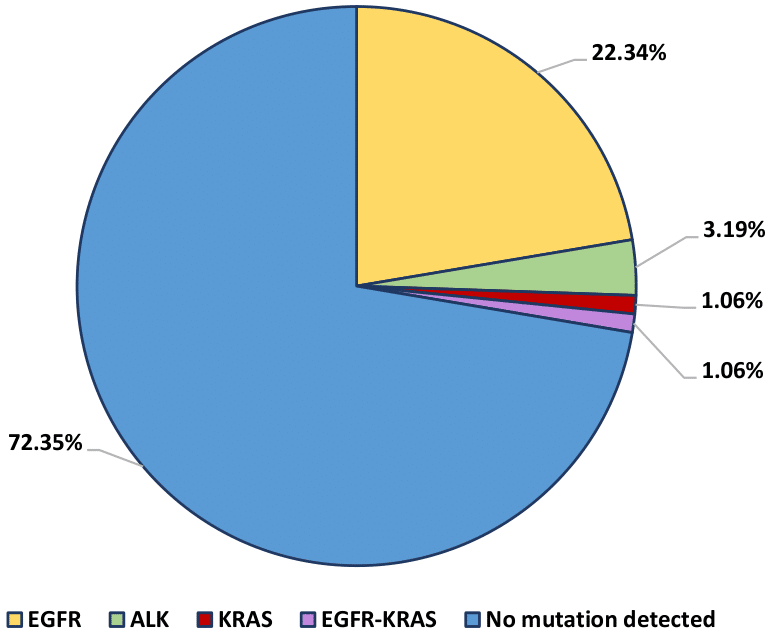
frequency of driver mutations in lung adenocarcinoma patients

**Driver gene mutations:** among the 94 samples analyzed, we identified specific mutations in the EGFR and KRAS genes, as well as a fusion of the ALK gene. EGFR gene mutations were detected in 21 cases, representing 22.34% of the total sample. A single KRAS gene mutation was identified (1.06%), and ALK gene fusion was detected in 3 patients (3.19%). Furthermore, one case presented two concurrent mutations, namely an exon 19 deletion in the EGFR gene and a KRAS gene mutation (Figure 1).

Among the 94 samples analyzed, no mutations were identified in the other tested genes, including ROS1, RET, MET, NTRK1, NTRK2, NTRK3, HER2, BRAF, as well as in other exons of the EGFR and KRAS genes. These results reveal an absence of significant genetic alterations in these genes among patients with NSCLC in our cohort (Table 1).

**Table 1:** driver gene mutation in our cohort

Mutant gene	Gender	Mutation rate (%, n=94)
EGFR	M = 11, F = 10	21 (22.34%)
ALK	M = 0, F = 3	3 (3.19%)
KRAS	M =1, F = 0	1 (1.06%)
ROS1	M = 0, F = 0	0 (0.00%)
BRAF	M = 0, F = 0	0 (0.00%)
HER2	M = 0, F = 0	0 (0.00%)
RET	M = 0, F = 0	0 (0.00%)
MET	M = 0, F = 0	0 (0.00%)
NTRK1	M = 0, F = 0	0 (0.00%)
NTRK2	M = 0, F = 0	0 (0.00%)
NTRK3	M = 0, F = 0	0 (0.00%)
EGFR-KRAS	M = 1, F = 0	1 (1.06%)

M: male; F: female

**Figure 2:**
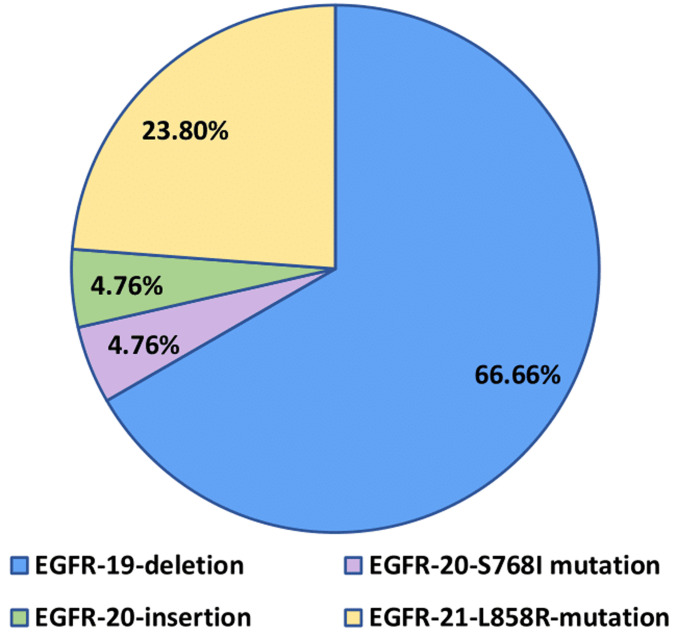
frequency of Epidermal Growth Factor Receptor (EGFR) sub-type mutations in advanced lung adenocarcinoma patients

**Epidermal Growth Factor Receptor (EGFR) gene mutations:** among the 21 cases with EGFR gene mutations, various variations were identified. Exon 19 deletions were present in 66.66% of cases (14 out of 21), representing the most frequent mutation. An exon 20 S768I mutation was observed in 4.76% of cases (1 out of 21), an exon 20 insertion was detected in another patient, also at a rate of 4.76% (1 out of 21), and exon 21 L858R mutations were identified in 23.80% of patients (5 out of 21) (Figure 2). No cases with concurrent EGFR gene mutations were identified (Table 2).

**Table 2:** Epidermal Growth Factor Receptor (EGFR) gene mutation

Mutant gene	Gender	Mutation rate (%, n=94)	Percentage/EGFR mutation (%, n=21)
EGFR-19-deletion	M = 8, F = 6	14 (14.89%)	14 (66.66%)
EGFR-20-S768I mutation	M = 1, F = 0	1 (1.06%)	1 (4.76%)
EGFR-20-insertion	M = 1, F = 0	1 (1.06%)	1 (4.76%)
EGFR-21-L858R-mutation	M = 1, F = 4	5 (5.31%)	5 (23.80%)

M: male; F: female

**Correlation between mutation status and clinical characteristics:** in the study's results, it was observed that the rate of positive mutations was slightly higher in women, with a percentage of 54.16%, compared to men (45.84%). However, apart from this data regarding gender and histological type, no other detailed information could be obtained for the patients included in the study. It is important to note that the analysis of other potential factors, such as age, smoking history, medical history, the presence of metastases, or other relevant clinical characteristics, was not conducted due to the lack of availability of this data.

## Discussion

According to the latest global cancer data published in 2020, the number of new cancer cases reached 18 million, with cancer-related deaths totaling 9.8 million worldwide. Lung cancer ranks first in terms of incidence and mortality in men and second in women after breast cancer. In the same year, in Morocco, lung cancer led to 6,551 deaths, making it the leading cause of cancer-related mortality. In terms of incidence, it ranks second among the most diagnosed cancers, just after breast cancer in women, with a total of 7,353 cases reported [6].

Non-small cell lung cancer is a complex and heterogeneous disease that requires a personalized approach based on the specific molecular profile of each patient. However, in Morocco, the understanding of this molecular profile remains incomplete due to various constraints. On the one hand, the lack of financial resources dedicated to oncology and molecular biology research poses a major obstacle: the high costs of advanced equipment, genetic tests, and molecular analyses have limited the capacity of Moroccan researchers to conduct in-depth studies on NSCLC. On the other hand, the lack of suitable technological infrastructure has also hindered the conduct of large-scale studies on the genetic mutations associated with this disease. Specialized laboratories equipped with cutting-edge genome analysis technologies are scarce in Morocco, making it difficult to conduct comprehensive and extensive studies on NSCLC. As a result, the country lacks robust scientific data regarding the molecular profile of NSCLC, which directly impacts treatment choices for patients. Targeted therapies and immunotherapy, which have demonstrated their effectiveness in certain subtypes of NSCLC, may not be fully utilized in the absence of precise genetic information. This situation is even more concerning as lung cancer is on the rise in Morocco due to risk factors such as smoking, air pollution, and occupational exposure.

For the optimal management of patients with NSCLC, a better understanding of the molecular profile of the disease and its impact on prognosis and treatment response is therefore required. Our study aims to address this gap in research on the molecular profile of NSCLC in Morocco, despite having a relatively limited sample size and incomplete clinical data. We seek to identify specific genetic mutations associated with this disease in Moroccan patients, to the best of our ability. While our results cannot be generalized to the entire population of NSCLC patients in Morocco, our goal is to contribute to the creation of a robust genetic database specific to Moroccan NSCLC, which could serve as a reference for future research and the development of more personalized and effective treatment strategies for Moroccan patients with this disease.

Cytological samples have proven to be highly effective as a genetic detection method for implementing targeted therapy for NSCLC patients. Our results revealed unique mutation rates in the EGFR, KRAS, and ROS1 genes among non-small cell lung cancer patients. We noted that 22.34% of patients had mutations in the EGFR gene, 3.19% in the ALK gene, and 1.06% in the KRAS gene. Among the patients studied, only one patient had a co-mutation of the EGFR and KRAS genes, accounting for 1.06%, indicating the simultaneous presence of an exon 19 deletion in the EGFR gene and a KRAS gene mutation.

Epidermal Growth Factor Receptor (EGFR) is a transmembrane receptor widely distributed in various cells, including mammalian epithelial cells and fibroblasts. This suggests a relationship between the EGFR signaling pathway and important processes such as cell proliferation, metastasis, angiogenesis (formation of new blood vessels), and apoptosis (programmed cell death) in the context of cancer.

In our study, we observed an EGFR mutation rate of 22.34%, highlighting a prevalence higher than that observed in the European Caucasian population, which generally ranges from 10% to 15% [7], as well as the Egyptian population, where the rate is 12.5% according to the study by Ismail MS *et al*. [8]. However, it is worth noting that this rate is significantly lower than that observed in Asia, ranging from 30% to 50% as reported in reference studies [9-12]. Likewise, it is lower than the rate observed in the Brazilian population, which reaches 42% according to the results of Palacio S *et al*. [13], as well as the rate found in the Indian population, which is 27.53%, as documented by Nakra T *et al*. [14].

Our results are consistent with other studies recruiting Moroccan patients, approaching the 22% rate identified in the study conducted by Lemine Sow M *et al*. [15] (Table 3).

**Table 3:** comparison of Epidermal Growth Factor Receptor (EGFR) mutation rates across different regions and studies

Studies	Region	Rate of EGFR mutation (%)	Year
Our study	Morocco	22.34%	2023
Sow ML *et al*.	Morocco	22%	2021
Shen F *et al*.	Asia	47%	2023
Han B *et al*.	Asia	49.3%	2017
Zhou Y *et al*.	Asia	34.9%	2017
Zhang Y *et al*.	China	38.4%	2016
	Japan	36.6%	
	Korea	32.4%	
	North and South America	24.4%	
	Europe	14.1%	
	Afro-American	17.2%	
Barlesi F *et al*.	Europe	11%	2016
Palacio S *et al*.	Brazil	42%	2019
Kassem M *et al*.	Egypt	12.5%	2023
Nakra T *et al*.	India	27.53	2020

**Figure 3:**
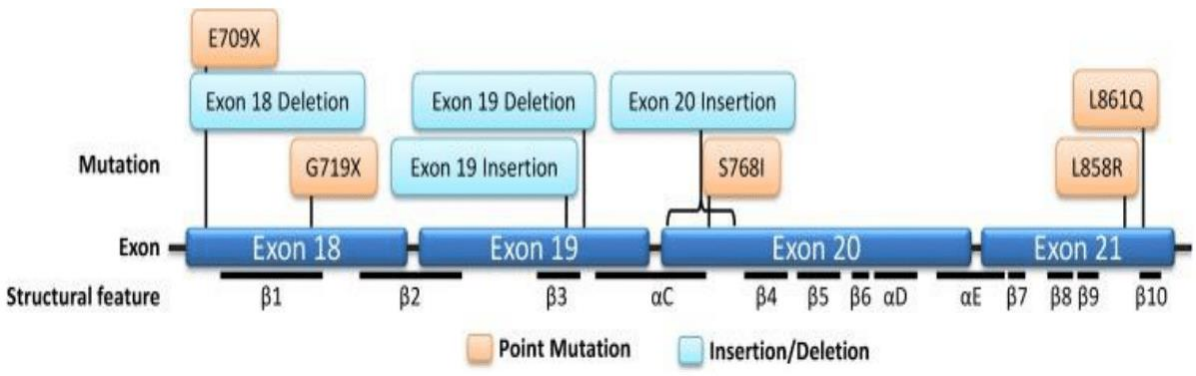
lollipop plot showing the position of Epidermal Growth Factor Receptor (EGFR) mutations and structural features of EGFR

These differences in mutation rates can be attributed to genetic and environmental factors specific to each population. Ethnic and geographical variations may also play a role in the prevalence of EGFR mutations in these patient groups. Among patients with lung adenocarcinoma, the most common mutations affecting the EGFR gene can be classified into four types: point mutations in exon 18, deletions in exon 19, insertions in exon 20, and point mutations in exon 21 [16] (Figure 3). In that figure, orange boxes indicate point mutations; blue boxes indicate insertion/deletion mutations [17].

**Figure 4:**
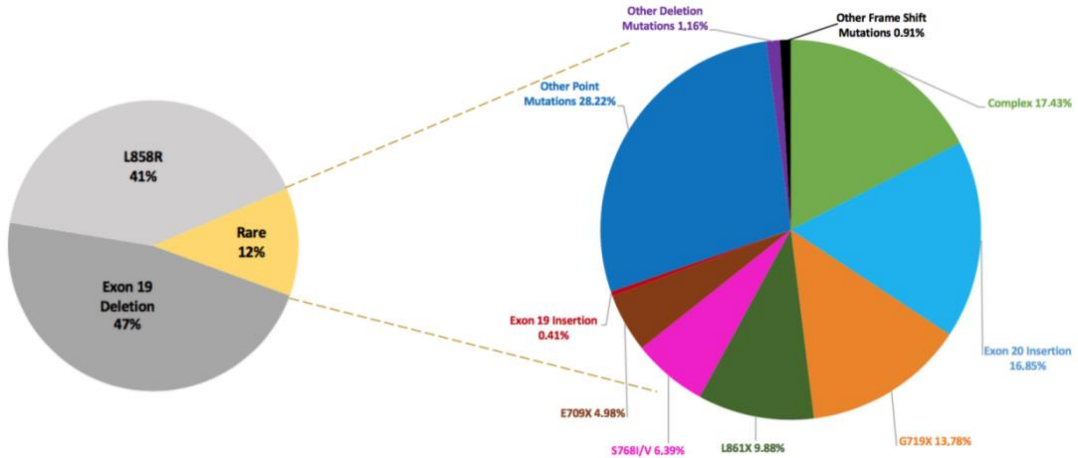
pie chart showing the frequencies of Epidermal Growth Factor Receptor (EGFR) mutations in non-small cell lung cancer (NSCLC)

Approximately 90% of these four types consist of EGFR-19-Deletion and EGFR-21-L858R-mutation [17], known as classical activating mutations, and are well-established as strong predictors of a favorable clinical response to EGFR tyrosine kinase inhibitors (Figure 4). In Figure 4, data was acquired from the Catalogue of Somatic Mutations in Cancer (COSMIC) databases. Data was filtered by Harrison P *et al*. to contain only mutations from adenocarcinoma. The common resistance mutations T790M and C797S were filtered out [17].

These two mutations also account for 90.46% of the observed EGFR mutations in our study. These findings align with the conclusions of other studies conducted on diverse populations [10-15,18-20]. These studies also found that these two mutations are the most prevalent (Table 4), underscoring the reliability of our observations and confirming the significance of these mutations in treatment response among patients with adenocarcinoma of the lung.

**Table 4:** prevalence of Epidermal Growth Factor Receptor (EGFR) Exon 19 deletion and Exon 21 L858R mutations in various regions worldwide

Studies	Region	Exon 19-deletion	Exon 21-L858R-mutation
Zhang Y *et al*.	Asia	32.3%	32.3%
Zhou Y *et al*.	Asia	40%	30%
Palacio S *et al*.	Brazil	54%	28%
Nakra T *et al*.	India	56.6%	29.8%
Sow ML *et al*.	Morocco	65.8%	17.8%
Cote ML	USA	58.3%	25%
Ramadhan H *et al*.	Iraq	65.8%	26.3%
Yang M *et al*.	China	42.8%	36.36%

Beyond EGFR, our study also assessed the mutation rates in other reference genes, including KRAS, ALK, ROS1, HER2, RET, MET, NTRK1, NTRK2, and NTRK3, which play essential roles in regulating cell growth and tumor progression in patients with lung adenocarcinoma. We detected one KRAS gene mutation in a single patient (1.06%), while ALK gene fusion was observed in three patients (3.19%), in addition to one case with concurrent mutations, namely, an EGFR exon 19 deletion and a KRAS gene mutation. No mutations were identified in the ROS1, HER2, RET, MET, NTRK1, NTRK2, and NTRK3 genes in our cohort. Although these mutation rates may seem relatively low, it's important to note that these mutations are rare and often specific to certain patient subgroups.

The significance of the KRAS gene in lung cancer has been extensively studied, and mutations in this gene are frequently associated with smoking. In our study, we detected a single KRAS gene mutation, corresponding to a rate of 1.06%. These results align with the literature, which shows that KRAS mutations are less frequent than EGFR mutations but are more common among patients with NSCLC who have a history of smoking. Regarding the ALK gene, our study revealed an ALK gene fusion in three patients, representing a rate of 3.19%. ALK fusions are associated with specific patient subgroups, including younger patients and non-smokers. In our cohort, we detected a relatively high prevalence of ALK gene fusion, which could indicate a higher proportion of non-smokers or younger patients in our sample.

As for the other reference genes such as ROS1, HER2, RET, MET, NTRK1, NTRK2, and NTRK3, our study did not identify mutations in these genes in patients with lung adenocarcinoma. These results are consistent with the fact that these mutations are rare and may be specific to particular patient subgroups. Despite the low mutation rate observed in these genes, it's important to note that detecting these mutations could be clinically significant for certain patients as they could guide the choice of targeted treatment.

The analysis of other clinical factors such as age, smoking history, medical history, and the presence of metastases was not conducted in our study due to the limited number of cases and the lack of complete clinical data. However, despite these limitations, our study contributes to filling a gap in research on the molecular profile of NSCLC in Morocco by providing initial insights into specific genetic mutations in a group of Moroccan patients with this disease.

## Conclusion

Lung cancer remains a major public health challenge, with high diagnosis rates and substantial mortality worldwide. Lung adenocarcinoma, the most common subtype of lung cancer, requires more targeted treatment approaches to improve patient survival. Our study, albeit limited in terms of the number of cases and clinical data, provides valuable insights into the molecular profile of non-small cell lung cancer (NSCLC) in Morocco. We observed varied rates of genetic mutations, with a significant prevalence of EGFR mutations, particularly exon 19 deletions and exon 21 mutations, which are associated with a better response to EGFR tyrosine kinase inhibitors. Additionally, we identified KRAS gene mutations and ALK gene fusions, although the latter were less frequent. Despite challenges related to financial resources and technological infrastructure, our study contributes to the accumulation of data on the molecular profile of lung cancer in Morocco. These findings serve as a foundation for future research and the development of more personalized treatment strategies for Moroccan patients with this disease. However, it is essential to continue research in this field and expand studies to larger samples with more comprehensive clinical data. This will lead to a better understanding of the molecular epidemiology of lung cancer in Morocco and the optimization of therapeutic choices for patients. Ultimately, the goal is to enhance the management of this complex and heterogeneous disease by providing more targeted and effective care to Moroccan patients with lung adenocarcinoma.

### What is known about this topic

- *Non-small cell lung cancer (NSCLC) is characterized by genetic heterogeneity, with various genetic alterations, and understanding the prevalence of these genetic alterations is crucial to guide personalized treatment approaches and improve clinical outcomes for patients;*
- *The variation in the prevalence of gene mutations associated with NSCLC among different ethnic groups and geographical regions suggests potential variations in the molecular landscape of this disease based on ethnicity and geography;*
- *Certain genetic mutations, such as those affecting the ALK, KRAS, and BRAF genes, are also implicated in NSCLC, although less frequently than EGFR mutations*.

### What this study adds

- *This study enabled the establishment of a molecular profile of NSCLC in a group of Moroccan patients through a simultaneous analysis of a set of genes related to this disease, thereby identifying and detecting co-mutations;*
- *The study highlighted a relatively high prevalence of EGFR mutations among Moroccan patients with NSCLC, compared to the Caucasian population, and lower than that observed in Asian populations, additionally, it confirmed previous findings regarding EGFR and ALK mutations in Moroccan patients;*
- *The study confirmed that exons 19 and 21 of the EGFR gene are the most frequently mutated sites in non-small cell lung cancer*.
